# Influence of Ketorolac Supplementation on Pain Control for Knee Arthroscopy: A Meta‐Analysis of Randomized Controlled Trials

**DOI:** 10.1111/os.12608

**Published:** 2020-02-19

**Authors:** Rui‐jie Wan, Shao‐fan Liu, Zhi‐ping Kuang, Qiang Ran, Chen Zhao, Wei Huang

**Affiliations:** ^1^ Department of Orthopaedic Surgery the First Affiliated Hospital of Chongqing Medical University Chongqing China; ^2^ Department of Orthopaedic Surgery Chongqing Traditional Chinese Medical Hospital Chongqing China

**Keywords:** ketorolac supplementation, knee arthroscopy, meta‐analysis, pain control, randomized controlled trials

## Abstract

**Introduction:**

The efficacy of ketorolac supplementation on pain control for knee arthroscopy remains controversial. We conduct a systematic review and meta‐analysis to explore the impact of ketorolac supplementation on pain intensity after knee arthroscopy.

**Methods:**

We search PubMed, EMbase, Web of science, EBSCO, and Cochrane library databases through September 2018 for randomized controlled trials (RCTs) assessing the effect of ketorolac supplementation *vs* placebo on pain management after knee arthroscopy. This meta‐analysis is performed using the random‐effect model.

**Results:**

Ten RCTs involving 402 patients are included in the meta‐analysis. Overall, compared with control group for knee arthroscopy, ketorolac supplementation is associated with notably reduced pain scores at 1 h (*MD* = −0.66; 95% *CI* = −1.12 to −0.21; *P* = 0.004) and 2 h (*MD* = −0.90; 95% *CI* = −1.74 to −0.07; *P* = 0.03), prolonged time for first analgesic requirement (*MD* = 1.94; 95% *CI* = 0.33 to 3.55; *P* = 0.02) and decreased number of analgesic requirement (*RR* = 0.41; 95% *CI* = 0.23 to 0.75; *P* = 0.003), but has no obvious impact on analgesic consumption (*MD* = −0.56; 95% *CI* = −1.14 to 0.02; *P* = 0.06), as well as nausea and vomiting (*RR* = 0.44; 95% *CI* = 0.12 to 0.21; *P* = 0.21).

**Conclusions:**

Ketorolac supplementation is effective to produce pain relief for knee arthroscopy.

## Introduction

Knee arthroscopy has been widely accepted as the most important method to diagnose and treat knee diseases, and is characterized by sound diagnosis and minimal invasion during the surgery^1^, ^2^, ^3^. Arthroscopic surgery of the knee is preferred by the majority of properly selected and well‐informed patients^4^, ^5^, ^6^. Postoperative stay after the surgery is significantly shorter in patients receiving local anesthesia than general anesthesia^7^. However, a significant number of patients encounter the moderate to severe pain 24 h after knee arthroscopy, and this pain may become worst and affect patients’ sleep and activity levels^8^, ^9^. In addition, early recovery of these patients is significantly hindered by the obvious pain which can further increase the total cost of such procedures^10^.

The presentation of pain after arthroscopic surgery is determined by the procedure of surgery and invasive procedures can result in moderate to severe pain^11^, ^12^. In order to provide better pain management after knee arthroscopy, many drugs (e.g. morphine and bupivacaine) have been developed to reduce postoperative pain intensity^13^, ^14^, ^15^. Analgesic opioids are used widespread to control moderate and severe postoperative pain, but they do not alleviate patient discomfort and result in side effects in the dose‐dependent method^16^, ^17^. Nonsteroidal anti‐inflammatory drugs (NSAIDs) have been reported to reduce postoperative pain via intra‐articular injection. Intra‐articular analgesia offer important potential in reducing postoperative disability, preventing the onset of pain, and avoiding the need for additional drugs. It may reach good analgesia in the immediate postoperative period by the administration of analgesic drugs^18^, ^19^.

NSAIDs such as ketorolac administered intra‐articularly provide good postoperative pain relief after the surgery^19^. Ketorolac has a high affinity with protein, and produces the analgesic effect through harnessing the production of prostaglandins^20^, ^21^. Ketorolac is reported to control mild to severe pain observed after certain kinds of surgical procedures, and has comparable analgesic effect and longer duration compared to opioid drugs^22^. Advantages of ketorolac over narcotic analgesics include it not producing depression in the respiratory and central nervous systems, and its more favorable safety profile^23^.

However, the efficacy of ketorolac supplementation on pain control after knee arthroscopy has not been well established. Recently, several studies on the topic have been published, and the results have been conflicting^18^, ^19^, ^24^, ^25^, ^26^. With accumulating evidence, we therefore perform a systematic review and meta‐analysis of RCTs to investigate the efficacy of ketorolac supplementation *vs* placebo on pain management after knee arthroscopy.

## Materials and Methods

Ethical approval and patient consent are not required because this is a systematic review and meta‐analysis of previously published studies. The systematic review and meta‐analysis are conducted and reported in adherence to PRISMA (Preferred Reporting Items for Systematic Reviews and Meta‐Analyses)^27^.

### *Study Eligibility Criteria (PICOS)*

The inclusive selection criteria are as follows: (i) participants (P): patients undergoing knee arthroscopy; (ii) intervention (I): ketorolac supplementation; (iii) control (C): placebo; (iv) outcomes (O): the primary outcomes are pain scores at 1 h and 2 h; secondary outcomes include time for first analgesic requirement, number of analgesic requirement, analgesic consumption, nausea and vomiting; (v) study design (S): RCT.

### *Exclusion Criteria*

The exclusion criteria include: (i) the history of using analgesics 24 h before surgery; (ii) the history of bleeding or coagulation problems during the last month before surgery; (iii) renal and liver failure; (iv) severe cardiopulmonary disease; (v) coagulopathy; (vi) intolerance or contraindications to ketorolac; (vii) pregnancy and lactation; and (viii) a history of drug and alcohol abuse.

### *Search Strategy and Study Selection*

Two investigators have independently searched the following databases (inception to September 2018): PubMed, EMbase, Web of science, EBSCO, and Cochrane library databases. The electronic search strategy is conducted using the combination keywords: “ketorolac” and “knee arthroscopy”. We also checked the reference lists of the screened full‐text studies to identify other potentially eligible trials.

### *Data Extraction and Outcome Measures*

We have extracted the following information: author, number of patients, age, gender, body weight and detail methods in each group. Data have been extracted independently by two investigators, and discrepancies are resolved by consensus. We also contact the corresponding author to obtain the data when necessary.

### *Quality Assessment in Individual Studies*

Methodological quality of the included studies is independently evaluated using the modified Jadad scale^28^. There are three items for Jadad scale: randomization (0–2 points), blinding (0–2 points), dropouts and withdrawals (0–1 points). The score of Jadad Scale varies from 0 to 5 points. An article with Jadad score ≤2 is considered to be of low quality. If the Jadad score ≥3, the study is thought to be of high quality^29^.

### *Statistical Analysis*

We estimate the standard mean difference (*MD*) with 95% confidence interval (*CI*) for continuous outcomes (pain scores at 1 h and 2 h, time for first analgesic requirement, and analgesic consumption) and risk ratio (*RR*) with 95% CIs for dichotomous outcomes (number of analgesic requirement, nausea and vomiting). A random‐effects model is used regardless of heterogeneity. Heterogeneity is reported using the *I* ^2^ statistic, and *I* ^2^ > 50% indicates significant heterogeneity^30^. Whenever significant heterogeneity is present, we search for potential sources of heterogeneity via omitting one study in turn for the meta‐analysis or performing subgroup analysis. All statistical analyses are performed using Review Manager Version 5.3 (The Cochrane Collaboration, Software Update, Oxford, UK).

## Results

### *Literature Search, Study Characteristics and Quality Assessment*

**Figure 1:**
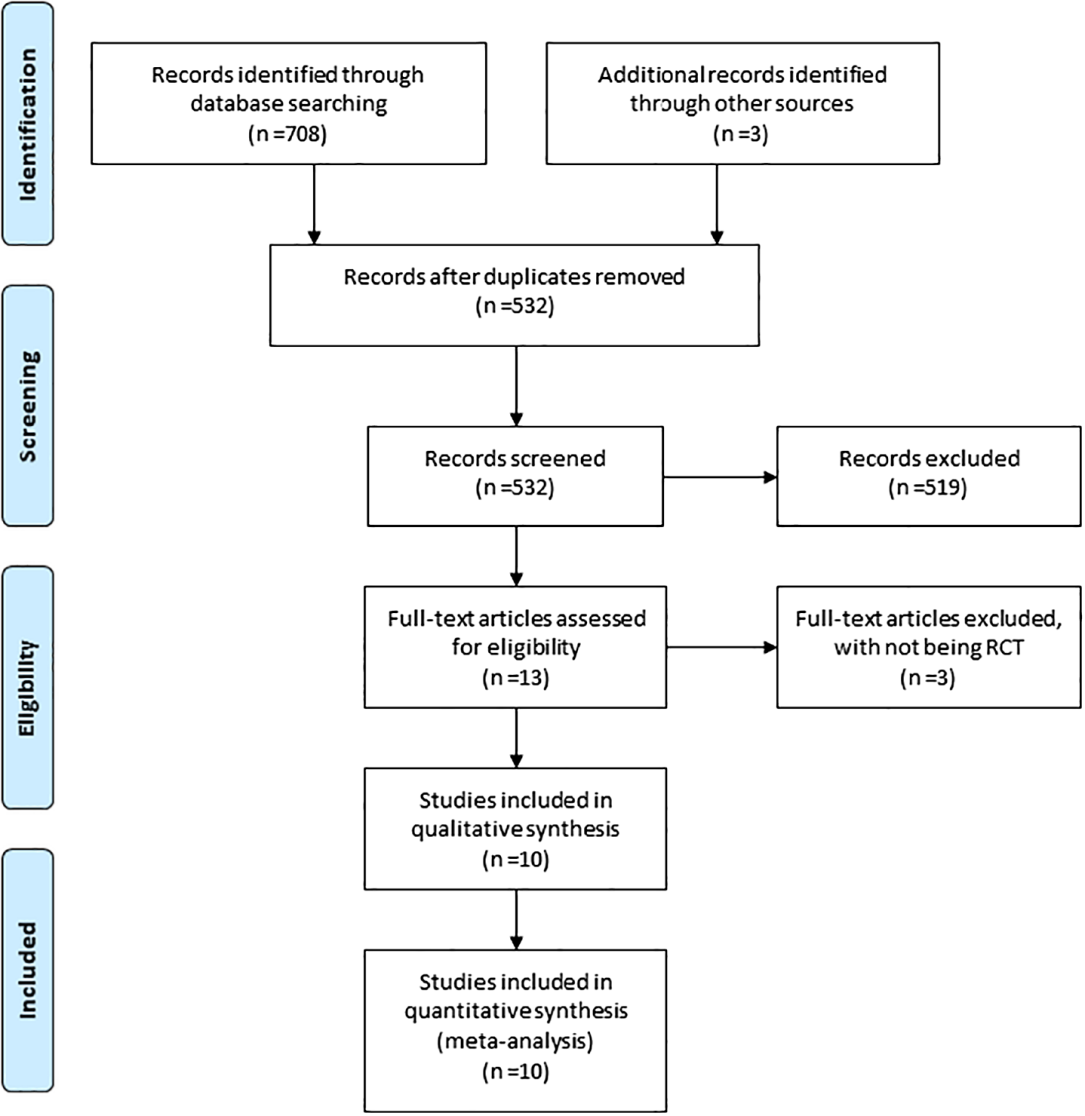
Flow diagram of study searching and selection process.

A detailed flowchart of the search and selection results is shown in Fig. 1. Seven hundred and seventy‐nine potentially relevant articles are identified initially. Two hundred and forty‐seven duplicates and 519 studies are removed after reading the titles/abstract. Three articles are excluded for not being RCT. Finally, ten RCTs that meet our inclusion criteria are included in the meta‐analysis^18^, ^19^, ^24^, ^25^, ^26^, ^31^, ^32^, ^33^, ^34^, ^35^.

The baseline characteristics of the 10 eligible RCTs in the meta‐analysis are summarized in Table 1. The 10 studies are published between 1992 and 2018, and sample sizes range from 30 to 60 with a total of 402. PICOS results are as follows: (i) participants (P): all patients undergo knee arthroscopy and have similar age, gender, body weight and operation time between two groups (*P* > 0.05); (ii) intervention (I): the ketorolac is administered by intra‐articular or intravenous approaches before, during or after the surgery, and its doses range from 5 mg to 60 mg. Four RCTs report ketorolac as the adjunctive analgesic to bupivacaine^18^, ^34^, ^35^ or ropivacain^25^; (iii) control (C): intra‐articular ropivacaine, bupivacaine or placebo; (iv) outcomes (O): among the 10 studies included here, two studies report pain scores at 1 h and 2 h^19^, ^34^, three studies report time for first analgesic requirement^25^, ^34^, ^35^, five studies report a number of analgesic requirements^19^, ^26^, ^31^, ^34^, ^35^, three studies report analgesic consumption^24^, ^34^, ^35^, and two studies report nausea and vomiting^31^, ^35^; and (v) study design (S): all studies are RCTs. Jadad scores of the 10 included studies vary from three to five, and all 10 studies are considered to be high‐quality ones according to quality assessment.

**Table 1:** Characteristics of included studies

No.	Author and year	Ketorolac group						Control group							
		Sample size	Age (years)	Female (*n*)	Body weight (kg)	Operation time (min)	Methods	Sample size	Age (years)	Female (n)	Body weight (kg)	Operation time (min)	Methods	Outcomes	Jada scores
1	Solheim 2018	22	51.0 ± 13.3	12	—	—	intra‐articular ketorolac (5 mg)	20	52.8 ± 12.1	11	—	—	placebo	analgesic consumption	4
2	Rokhtabnak 2015	20	45.05 ± 13.6	6	76.45 ± 9.08	39.45 ± 9.6	intra‐articular ketorolac (30 mg) and ropivacaine (150 mg) at the end of knee arthroscopic surgery	20	42.4 ± 12.2	3	83.35 ± 10.5	38.7 ± 9.7	intra‐articular ropivacaine (150 mg)	time for first analgesic requirement	5
3	Stalman 2009	20	41.7 ± 8.4	10	—	27.4 ± 9.7	2 mL of ketorolac 30 mg/mL in 8 mL of NaCl 9 mg/mL before surgery	20	44.5 ± 8.8	13	—	32 ± 15.9	placebo	number of analgesic requirement,	4
4	Rao 2005	30	32.66 ± 8.86	4	62.9 ± 11.35	—	10 ml of 0.25% bupivacaine, 1 ml (30 mg) of ketorolac and 9 rnl of saline intra‐articularly	30	32.5 ± 10.08	3	61.2 ± 10.25	—	10 rnl of intra‐articular saline and 10 rnl of 0.25% bupivacaine	—	3
5	Calmet 2004	20	—	—	—	—	postoperative injection of 60 mg intra‐articular ketorolac	20	—	—	—	—	placebo	pain scores at 1 h and 2 h, number of analgesic requirement	3
6	Gupta 1999	20	36.6 ± 15.1	6	—	—	60 mg intra‐articular ketorolac	20	44.3 ± 16.4	3	—	—	placebo	number of analgesic requirement, nausea and vomiting	4
7	Thwaites 1996	15	38.4 ± 14.5	5	—	—	intravenous ketorolac 60 mg 15 min after skin incision	15	34.3 ± 14.1	2	—	—	placebo	—	3
8	Thwaites 1995	15	33.2 ± 11.7	7	—	—	intravenous ketorolac 60 mg 15 min after skin incision	15	39.2 ± 14	4	—	—	placebo	—	3
9	Reuben 1995	20	41 ± 17	—	80 ± 22	50 ± 22	intra‐articular 0.25% bupivacaine (28 mL) with ketorolac (60 mg)	20	46 ± 17	—	70 ± 10	47 ± 16	intra‐articular 0.25% bupivacaine (30 mL)	pain scores at 1 h and 2 h, time for first analgesic requirement, number of analgesic requirement, analgesic consumption	4
10	Smith 1992	19	42 ± 12	8	77 ± 17	38 ± 15	systemic ketorolac (60 mg) and intraarticular 0.5% bupivacaine (30 mL)	21	33 ± 13	9	84 ± 22	33 ± 11	intraarticular 0.5% bupivacaine (30 mL)	time for first analgesic requirement, number of analgesic requirement, analgesic consumption, nausea and vomiting	4

### *Primary Outcomes: Pain Scores at 1 h and 2 h*

**Figure 2:**
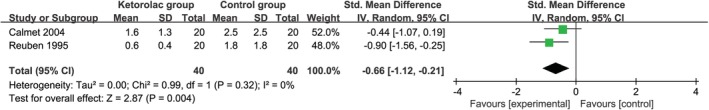
Forest plot for the meta‐analysis of pain scores at 1 h.

**Figure 3:**
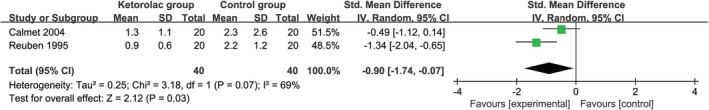
Forest plot for the meta‐analysis of pain scores at 2 h.

These outcome data are analyzed with the random‐effects model, and compared to control group for knee arthroscopy, ketorolac supplementation results in significantly reduced pain scores at 1 h (*MD* = −0.66; 95% *CI* = −1.12 to −0.21; *P* = 0.004) with no heterogeneity among the studies (*I* ^2^ = 0%, heterogeneity *P* = 0.32) (Fig. 2), and 2 h (*MD* = −0.90; 95% *CI* = −1.74 to −0.07; *P* = 0.03) with significant heterogeneity among the studies (*I* ^2^ = 69%, heterogeneity *P* = 0.07) (Fig. 3).

### *Sensitivity Analysis*

Significant heterogeneity is observed among the included studies for the pain scores at 2 h. Because there are just two RCTs included for the analysis of primary outcomes, we do not perform sensitivity analysis via omitting one study in order to detect the heterogeneity.

### *Secondary Outcomes*

**Figure 4:**
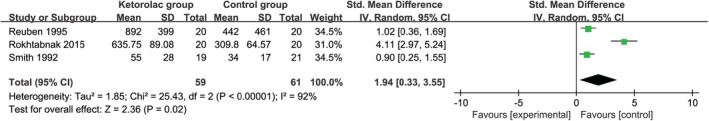
Forest plot for the meta‐analysis of time for first analgesic requirement (min).

**Figure 5:**
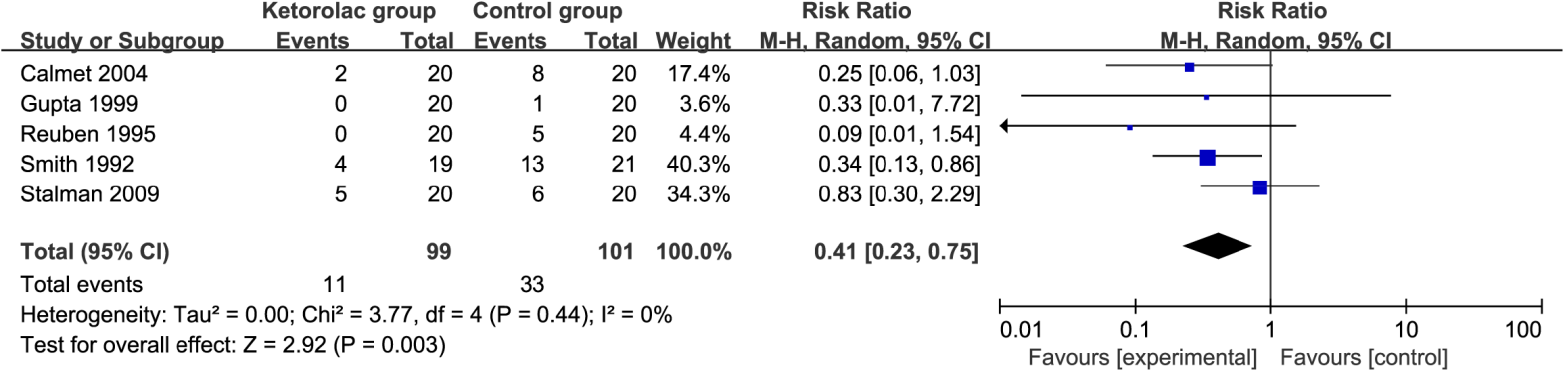
Forest plot for the meta‐analysis of number of analgesic requirement.

**Figure 6:**
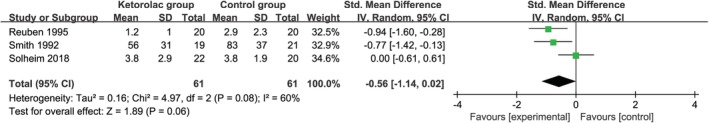
Forest plot for the meta‐analysis of analgesic consumption.

**Figure 7:**
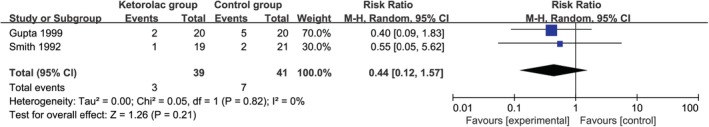
Forest plot for the meta‐analysis of nausea and vomiting.

In comparison with control group for knee arthroscopy, ketorolac supplementation is associated with remarkably longer time for first analgesic requirement (*MD* = 1.94; 95% *CI* = 0.33 to 3.55; *P* = 0.02; Fig. 4) and decreased number of analgesic requirement (*RR* = 0.41; 95% *CI* = 0.23 to 0.75; *P* = 0.003; Fig. 5), but shows no important impact on analgesic consumption (*MD* = −0.56; 95% *CI* = −1.14 to 0.02; *P* = 0.06; Fig. 6), as well as nausea and vomiting (*RR* = 0.44; 95% *CI* = 0.12 to 0.21; *P* = 0.21; Fig. 7).

## Discussion

Our meta‐analysis suggests that compared to control intervention for knee arthroscopy, ketorolac supplementation can favorably reduce pain scores at 1 h and 2 h, prolong the time for first analgesic requirement, and decrease the number of analgesic requirements, with no significant influence on analgesic consumption. Regarding the sensitivity analysis, there is significant heterogeneity for the pain scores at 2 h. One included RCT reports postoperative injection of 60 mg intra‐articular ketorolac *vs* placebo for pain relief^19^, whereas the other included RCT involves intra‐articular 0.25% bupivacaine (28 mL) with ketorolac (60 mg) *vs* intra‐articular 0.25% bupivacaine (30 mL)^34^. These indicate that the significant heterogeneity may be caused by the different combination of ketorolac, and the combination of ketorolac and bupivacaine may have synergistic effects for pain management.

Multimodal pain therapy has been strongly recommended for treatment of postoperative pain^36^, ^37^, and is theoretically supported by the additive or synergistic effects between different analgesics, and concomitant reduction of side effects because of lower doses of analgesics^38^. For instance, ketorolac combined with morphine and ropivacaine is found to give a synergistic effect for pain relief after arthroscopic procedures^31^. In one RCT, combining ketorolac and ropivacaine shows the beneficial effects on pain intensity, especially the pain on the movement up to 24 h postoperatively^25^. In addition, ketorolac administered directly to sites is likely to produce high local tissue concentrations and leads to few systemic complications^39^. There are different risk factors related to nausea and vomiting after surgery, and the type of anesthesia and the use of narcotics are regarded as the main factors that contribute to these issues. NSAIDs is found to attenuate the incidence of nausea and vomiting after surgery as compared with opioids^40^. There is no increase in nausea and vomiting between ketorolac supplementation and control intervention based on the results of our meta‐analysis.

This meta‐analysis has several potential limitations. Firstly, our analysis is based on 10 RCTs, and all of them have a relatively small sample size (*n* < 100). Overestimation of the treatment effect was more likely in smaller trials compared with larger samples. Next, there is significant heterogeneity, and different doses, drug combination, and administration time of ketorolac may have some impact on the pooling results. Finally, some unpublished and missing data may lead to bias in the pooled effect.

### *Conclusion*

Ketorolac supplementation can provide important benefits for pain control after knee arthroscopy.
